# Healthcare utilization for inpatient rehabilitation among people with traumatic brain injury before the introduction of the specialized rehabilitation hospital system: a nationwide retrospective cohort study

**DOI:** 10.3389/fneur.2025.1674199

**Published:** 2025-11-21

**Authors:** Suk Won Bae, Jiyoung Shin, Wha Me Park, Jeongha Kim

**Affiliations:** 1 Health Insurance Research Institute, National Health Insurance Service, Wonju, Republic of Korea; 2 The Institute for Occupational Health, Yonsei University College of Medicine, Seoul, Republic of Korea; 3 Graduate School of Public Health, Yonsei University, Seoul, Republic of Korea

**Keywords:** traumatic brain injury, inpatient rehabilitation, specialized rehabilitation hospital system, length of stay, healthcare delivery systems

## Abstract

**Introduction:**

In this study, we aimed to examine the use of inpatient rehabilitation services among individuals who experienced a traumatic brain injury (TBI) in 2017, before the establishment of the specialized rehabilitation hospital system.

**Methods:**

We analyzed healthcare resource utilization among patients hospitalized with TBI at acute care hospitals in 2017. Data were obtained from the National Health Insurance Service-Nationwide Health Insurance Database of South Korea. For patients who received inpatient rehabilitation, the length of stay (LOS) over the 2 years following their initial hospital admission was analyzed. Negative binomial regression was used to identify factors influencing LOS.

**Results:**

Patients who received inpatient rehabilitation were hospitalized for a mean of 145.2 days during the follow-up period [median: (Q1–Q3): 35 (16–141) days]. LOS was shorter in tertiary hospitals, general hospitals, primary hospitals, and clinics than in long-term care hospitals (LTCHs) (all *p* < 0.001). After discharge from the initial healthcare institution, the most common first transfer destination was LTCH (27.5%), followed by general and primary hospitals. For subsequent hospitalizations, approximately half (48.3%) of the patients were admitted to LTCHs.

**Conclusion:**

These findings underscore the need to establish a specialized rehabilitation hospital system.

## 1 Introduction

Traumatic brain injury (TBI), also known as intracranial injury, refers to brain injury caused by external physical forces (1, 2), leading to physical, cognitive, social, emotional, and behavioral impairments (3). Approximately 50–60 million people worldwide sustain TBI each year, resulting in an estimated global economic loss of about USD 400 billion annually (4), making TBI a major global public health concern (5–7). Patients with TBI require long-term treatment, including intensive care and rehabilitation (5, 8). Rehabilitation for TBI typically occurs in three phases: early rehabilitation during the acute stage, intensive rehabilitation during the subacute stage, and community-based rehabilitation during the maintenance stage (9). To optimize treatment and promote recovery in patients with moderate-to-severe TBI who require inpatient rehabilitation (8, 10, 11), transfer to an acute rehabilitation hospital is necessary during the subacute stage following acute treatment.

In the United States, inpatient rehabilitation facilities (IRFs) are acute rehabilitation hospitals that provide intensive inpatient care after the acute stage, in accordance with regulations established by the Centers for Medicare and Medicaid Services (12, 13). At least 60% of admitted patients must have one of 13 qualifying conditions, including brain injury. Each patient must receive an intensive, multidisciplinary rehabilitation program consisting of at least 3 h of therapy per day on 5 of 7 consecutive days (13). In 2000, Japan implemented kaifukuki (convalescent) rehabilitation wards (KRWs), comparable with the IRF model used in the United States (14). Patients requiring assistance with activities of daily living after acute treatment for conditions such as TBI are eligible for admission to KRWs. Rehabilitation therapy is limited to a maximum of 3 h per day, and physical therapists determine discharge or transfer to home care once rehabilitation goals are achieved (15).

During the study period (before 2020), South Korea had no formal referral protocols for transferring patients to acute rehabilitation hospitals that provide intensive rehabilitation following acute treatment (16). In response, the specialized rehabilitation hospital system was introduced in 2020. Under this system, hospitals meeting specific requirements are designated as acute rehabilitation hospitals to provide intensive rehabilitation during the subacute stage for patients with seven qualifying conditions, including brain injury (17, 18). For patients with brain injury, admission to an acute rehabilitation hospital is permitted within 90 days after onset or surgery, and intensive rehabilitation can be provided for up to 180 days, with a maximum of 4 h per day (19).

The assessment of healthcare resource utilization following acute treatment and before the introduction of the specialized rehabilitation hospital system can provide foundational data for evaluating the system’s effectiveness (13). Several studies have examined whether patients with stroke received appropriate inpatient rehabilitation before the system’s implementation; however, to the best of our knowledge, no published reports have investigated healthcare delivery systems for patients with TBI.

In this study, we aimed to examine the utilization of inpatient rehabilitation services among individuals who experienced TBI in 2017, before the introduction of the specialized rehabilitation hospital system, using data from the Nationwide Health Insurance Database (NHID), a large-scale administrative dataset.

## 2 Materials and methods

### 2.1 Data source

In this study, we tracked healthcare resource utilization among patients hospitalized with TBI at acute hospitals (tertiary or general hospitals) in 2017. Data were obtained from the National Health Insurance Service (NHIS)-NHID of South Korea. South Korea operates an NHIS that covers its entire population. The NHIS is a mandatory social insurance program offering short-term health coverage (20, 21). The NHIS-NHID includes medical data generated by healthcare institutions, containing detailed information on individuals’ baseline characteristics (e.g., eligibility, insurance premiums, and mortality), medical service utilization, healthcare institution characteristics, and health screening results (21, 22). The NHIS provides anonymized data with no missing values to ensure patient privacy. Researchers can access the data only within the secure online environment provided by the NHIS. This study was approved by the Institutional Review Board of Seoul National University Hospital (IRB No. H-2507-100-1658). The requirement for informed consent was waived because the NHIS data were anonymized and de-identified.

### 2.2 Study population

**Figure 1:**
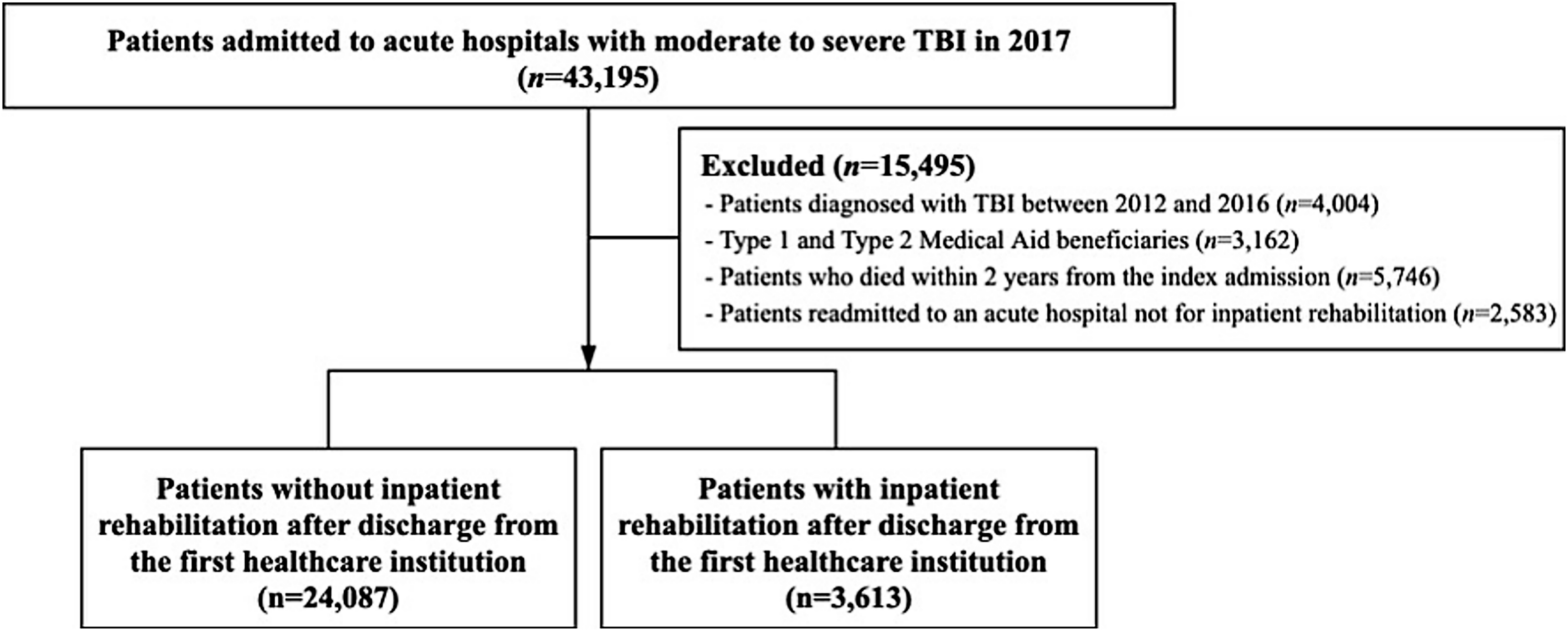
Schematic diagram of the study population.

The study population comprised 43,195 patients who were hospitalized for TBI at an acute care hospital as their first healthcare institution between January 1, 2017, and December 31, 2017. A washout period was set from 2012 to 2016, and 4,004 patients diagnosed with TBI during that period were excluded. TBI cases were identified using the International Classification of Diseases (10th Revision). Patients with a main or sub-diagnosis containing any of the following diagnosis codes were considered to have moderate-to-severe TBI and were also used to identify inpatient rehabilitation cases: cranial fracture (S02.0, S02.1, S02.7, S02.8, S02.9, S07.1, and T90.2), and intracranial injury (S06.1, S06.2, S06.3, S06.4, S06.5, S06.6, S06.7, S06.8, S06.9, and T90.5) (2, 11, 23). Because Medical Aid beneficiaries may have longer LOS due to lower hospitalization costs, 3,162 Medical Aid beneficiaries were excluded. Healthcare resource utilization was tracked for 24- month from each patient’s index admission date. In addition, 5,746 individuals who died during the follow-up period and 2,583 patients who were readmitted to an acute hospital for reasons other than inpatient rehabilitation (e.g., recurrence or complications) were excluded. A total of 27,700 patients were included in the final analysis. Figure 1 presents a schematic of the study population flow.

### 2.3 Variables

Patient age was categorized into four groups: <65, 65–74, 75–84, and ≥85 years.

The NHIS-NHID does not include a separate income variable; therefore, health insurance premium data were used as a proxy for patient income. Based on premium levels, patients were categorized into four quartiles, with the first quartile representing the lowest health insurance premium level (21).

Comorbidity was assessed using the Charlson Comorbidity Index (CCI). Patients were categorized into two groups based on their CCI scores: 0–1 indicated low comorbidity, while ≥2 indicated high comorbidity (24).

Because the NHIS-NHID does not include a dedicated severity variable, intensive care unit (ICU) admission was used as a proxy measure of clinical severity. Patients were categorized into two groups according to ICU admission during the medical care period. Those admitted to the ICU were considered to have higher severity, whereas those without ICU admission were regarded as having lower severity.

The healthcare institutions where patients received medical services were classified as tertiary hospitals, general hospitals, primary hospitals, long-term care hospitals (LTCHs), and clinics. The proportion of each type of healthcare institution was examined at each stage of hospitalization.

The LOS from the initial admission to an acute hospital for TBI during the follow-up period was the primary outcome variable in this study. A 24-month follow-up period was set to comprehensively capture inpatient rehabilitation utilization following the index admission (13). Transfers between hospitals were regarded as separate hospitalization episodes, rather than as continuous admissions. For patients with TBI who were admitted to multiple healthcare institutions during the follow-up period, the LOS was calculated as the sum of the LOS at each institution.

### 2.4 Statistical analysis

The sociodemographic characteristics of the group that did not receive inpatient rehabilitation (i.e., those who were not hospitalized during the follow-up period after discharge from the first healthcare institution) and the group that received inpatient rehabilitation (i.e., those who were readmitted to another healthcare institution for rehabilitation after discharge from the first healthcare institution) were compared using the chi-square test (*χ*^2^ test).

For the group that received inpatient rehabilitation, the LOS over the 2-year period following the initial admission to the first healthcare institution was analyzed using the Mann–Whitney U test and Kruskal–Wallis test to compare mean and median values across variables.

Furthermore, negative binomial regression was used to identify factors associated with LOS. Independent variables included age, sex, health insurance premium, CCI, ICU admission, and inpatient healthcare institution type. Multicollinearity among variables was evaluated using the variance inflation factor, and model fit was evaluated using the Akaike Information Criterion (AIC) and Bayesian Information Criterion (BIC).

In addition, the proportion of healthcare institutions where patients received medical care at each hospitalization stage was analyzed. For each stage, the denominator was defined as the number of patients who received medical care.

All statistical analyses were conducted using Statistical Analysis System software (version 9.4; SAS Institute, Cary, NC, USA).

## 3 Results

Table 1 presents the sociodemographic characteristics of patients with moderate-to-severe TBI who were admitted to an acute hospital for initial treatment, comparing those who did not receive inpatient rehabilitation after discharge from the first healthcare institution with those who were readmitted to another healthcare institution for inpatient rehabilitation. Of the 27,700 patients, 3,613 (13.0%) received inpatient rehabilitation after discharge, whereas 24,087 (87.0%) did not. The inpatient rehabilitation group included a greater proportion of patients aged ≥65 years, whereas the non-rehabilitation group had more patients aged <65 years (*p* < 0.001). A high CCI score (≥2) was observed in 45.7% of the non-rehabilitation group and 70.4% of the inpatient rehabilitation group (*p* < 0.001). In addition, 9.8% of patients in the inpatient rehabilitation group had a history of ICU admission, compared with 1.0% in the non-rehabilitation group (*p* < 0.001). The mean LOS at the first healthcare institution was 11.9 days for all patients, with a mean LOS of 20.7 and 10.6 days for the group that did and did not receive inpatient rehabilitation, respectively (Supplementary Table S1).

**Table 1:** Sociodemographic characteristics in the study population.

Variables	Total		Non-inpatient rehabilitation group^*^		Inpatient rehabilitation group^†^		*p*-value
	*N*	%	*N*	%	*N*	%	
Total	27,700	100.0	24,087	100.0	3,613	100.0	
Age							<0.001
<65	17,729	64.0	15,857	65.8	1,872	51.8	
65–74	4,531	16.4	3,828	15.9	703	19.5	
75–84	4,403	15.9	3,589	14.9	814	22.5	
≥85	1,037	3.7	813	3.4	224	6.2	
Sex							0.156
Male	18,697	67.5	16,221	67.3	2,476	68.5	
Female	9,003	32.5	7,866	32.7	1,137	31.5	
Health insurance premium							0.006
First quartile (lowest)	5,494	19.8	4,706	19.5	788	21.8	
Second quartile	5,671	20.5	4,957	20.6	714	19.8	
Third quartile	7,063	25.5	6,193	25.7	870	24.1	
Fourth quartile (highest)	9,472	34.2	8,231	34.2	1,241	34.3	
Charlson comorbidity index							<0.001
Low (0–1)	14,142	51.1	13,072	54.3	1,070	29.6	
High (≥2)	13,558	48.9	11,015	45.7	2,543	70.4	
Intensive care unit admission							<0.001
Yes	600	2.2	245	1.0	355	9.8	
No	27,100	97.8	23,842	99.0	3,258	90.2	

*Those who were not hospitalized during the follow-up period after discharge from the first healthcare institution.

^†^Those who were readmitted to another healthcare institution for rehabilitation after discharge from the first healthcare institution.

The group that received inpatient rehabilitation was hospitalized for a mean of 145.2 days during the follow-up period [median (Q1–Q3): 35 (16–141) days] (Table 2). Older and female patients had longer LOS (both *p* < 0.001). Patients with high CCI scores and those with a history of ICU admission also had longer LOS (both *p* < 0.001). Among the types of healthcare institutions, the mean LOS was longest in LTCHs (273.7 days, *p* < 0.001), followed by primary hospitals, general hospitals, tertiary hospitals, and clinics.

**Table 2:** Length of hospital stay in the inpatient rehabilitation group (*N* = 3,613).

Variables	Length of stay (day)		*p*-value^*^
	Mean (median)	Q1–Q3	
Total	145.2 (35)	16–141	
Age			<0.001
<65	106.9 (27)	13–78	
65–74	160.8 (40)	20–156	
75–84	190.4 (55)	23–260	
≥85	251.8 (110)	25–523	
Sex			<0.001
Male	135.6 (32)	16–121	
Female	166 (42)	19–186	
Health insurance premium			0.327
First quartile (lowest)	157.5 (37)	17–159.5	
Second quartile	134.9 (35)	17–135	
Third quartile	130.3 (34.5)	15–119	
Fourth quartile (highest)	153.7 (34)	16–157	
Charlson comorbidity index			<0.001
Low (0–1)	48.7 (18)	10–33	
High (≥2)	185.8 (54)	23–241	
Intensive care unit admission			<0.001
Yes	282.5 (141)	35–589	
No	130.2 (31)	15–113	
Inpatient healthcare institution			<0.001
Tertiary hospital	19.9 (11)	6–24	
General hospital	30.3 (21)	10–37	
Primary hospital	97.1 (25)	11–91	
Long-term care hospital	273.7 (148)	38–557	
Clinic	16 (13)	7–19	

*Mann–Whitney U test and the Kruskal–Wallis test.

Negative binomial regression analysis revealed several significant factors associated with LOS among patients who received inpatient rehabilitation (Table 3). Patients aged ≥85 years had longer LOS than those aged < 65 years (*p* = 0.004). Similarly, patients with high CCI scores and those with a history of ICU admission had longer LOS than their respective counterparts (both *p* < 0.001). The LOS was significantly shorter in tertiary hospitals, general hospitals, primary hospitals, and clinics than in LTCHs (all *p* < 0.001).

**Table 3:** Negative binomial regression model for the length of hospital stay in the inpatient rehabilitation group (*N* = 3,613).

Variables	Length of stay (day)			
	Adjusted model^*^			
	*β*	EXP (*β*)	SE	*p*-value
Age				
<65	1.00			
65–74	0.03	1.03	0.05	0.504
75–84	0.04	1.05	0.05	0.356
≥85	0.22	1.24	0.08	0.004
Sex				
Male	1.00			
Female	0.03	1.03	0.04	0.434
Health insurance premium				
First quartile (lowest)	0.01	1.01	0.05	0.809
Second quartile	−0.01	0.99	0.05	0.892
Third quartile	−0.07	0.93	0.05	0.102
Fourth quartile (highest)	1.00			
Charlson comorbidity index				
Low (0–1)	1.00			
High (≥2)	0.80	2.22	0.04	<0.001
Intensive care unit admission				
Yes	0.80	2.22	0.06	<0.001
No	1.00			
Inpatient healthcare institution				
Tertiary hospital	−2.28	0.10	0.06	<0.001
General hospital	−1.71	0.18	0.05	<0.001
Primary hospital	−0.76	0.47	0.05	<0.001
Clinic	−1.84	0.16	0.09	<0.001
Long-term care hospital	1.00			

*Statistically estimated from negative binomial regression analyses adjusted for all explanatory variables.

**Figure 2:**
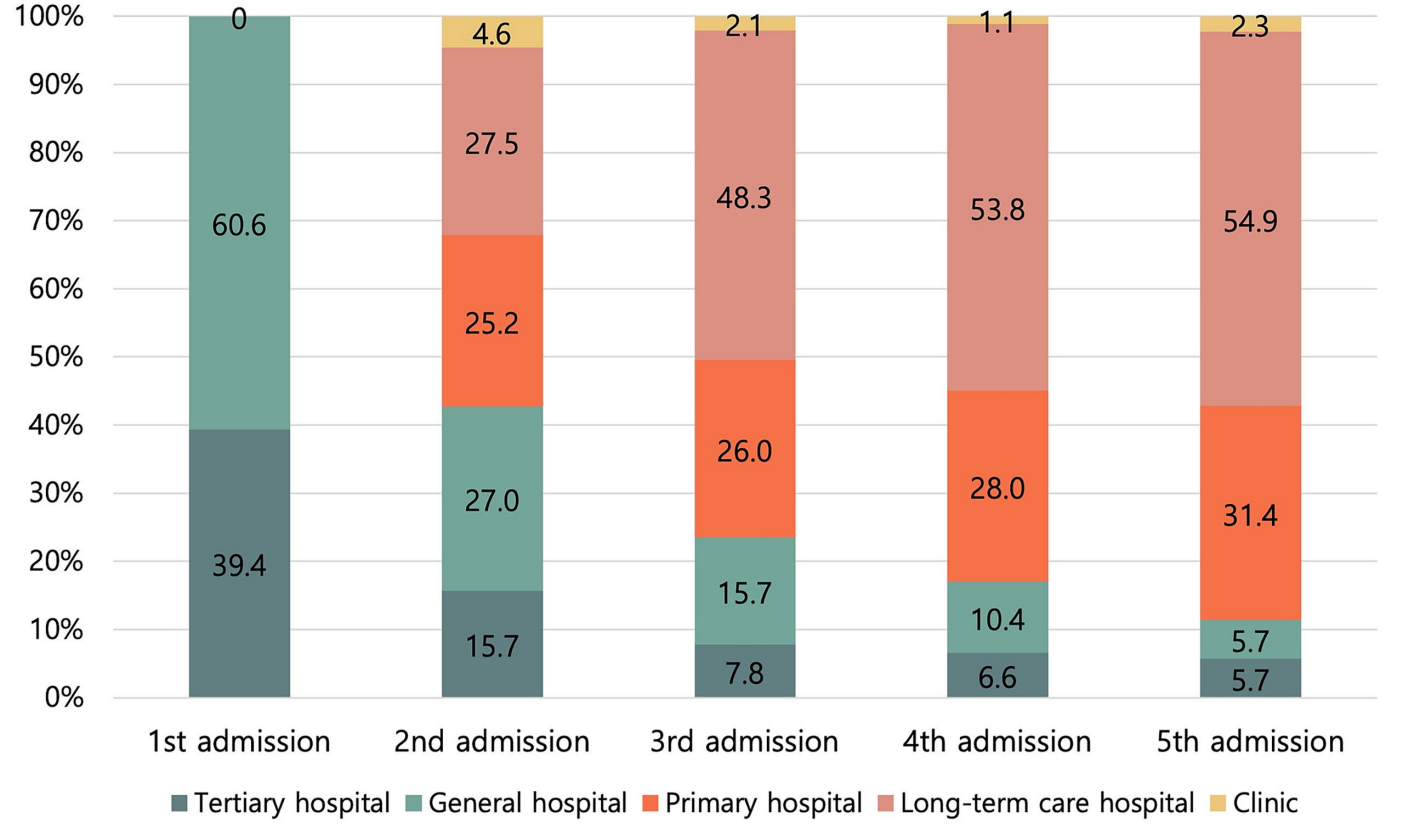
Proportions of each type of healthcare institution at each hospitalization stage.

Figure 2 shows the proportion of visits to each type of healthcare institution according to the order of hospitalization (from the first to the fifth). The number of patients is provided in Supplementary Table S2. After discharge from the initial healthcare institution, the most common destination for the first transfer was LTCHs (27.5%), followed by general and primary hospitals. During subsequent hospitalizations, approximately half (48.3%) of the patients were admitted to LTCHs. The average time to admission to a general hospital and an LTCH within 90 days after TBI was 21.9 days and 30.7 days, respectively (Supplementary Table S3).

## 4 Discussion

This is the first domestic study to examine the rehabilitation healthcare delivery system for patients with moderate-to-severe TBI before the introduction of the specialized rehabilitation hospital system in South Korea. Analysis of patient data from the Korean NHIS between 2017 and 2019 revealed that transfers to healthcare institutions capable of providing intensive rehabilitation following acute treatment were inadequate, largely due to the absence of formal referral protocols.

Of the 27,700 patients who received acute treatment for moderate-to-severe TBI in 2017, only 3,613 (13%) were transferred to another healthcare institution for inpatient rehabilitation, while most were discharged without receiving inpatient rehabilitation (Table 1). The group that did not receive inpatient rehabilitation had a mean LOS of 10.6 days at the acute treatment facility. In South Korea, outpatient-based rehabilitation services are rarely provided (18, 25), suggesting that rehabilitation for patients with TBI (non-inpatient rehabilitation group) was insufficiently delivered. A study on early rehabilitation and LOS among workers with moderate-to-severe work-related TBI in South Korea reported that 19.2% of patients received inpatient rehabilitation (11). Another study examining LOS associated with inpatient rehabilitation after ischemic stroke reported that 62.1% of all patients received inpatient rehabilitation (13). However, the stroke study included individuals admitted to Regional Cardio-Cerebrovascular Centers, whereas our study encompassed patients from all acute hospitals nationwide, including tertiary and general hospitals. This broader inclusion likely influenced the lower proportion of patients receiving inpatient rehabilitation after acute treatment. In South Korea, a comprehensive and standardized system that integrates patient severity and medical needs into the rehabilitation process was not implemented until 2020 (18, 26). Therefore, future studies should conduct more in-depth analyses to determine whether the low rate of inpatient rehabilitation primarily reflects differences in patient severity or the lack of formal referral protocols.

Following treatment at an acute hospital during the subacute stage after TBI, the most common first transfer destination was an LTCH. For subsequent transfers, approximately half of the patients (48.3%) were again admitted to an LTCH. The time to admission to an LTCH within 90 days after TBI was 30.7 days, while the average LOS at the first inpatient healthcare institution was 27.1 days. In South Korea, due to the limited availability of outpatient rehabilitation services and home-based care, as well as prevailing biases against outpatient rehabilitation, patients often experience prolonged hospital stays in LTCHs following acute treatment (26, 27). In South Korean LTCHs, rehabilitation therapy is provided; however, the typical duration is only 20–40 min per day, making intensive rehabilitation practically unfeasible (26, 28). South Korea’s rehabilitation services are reimbursed through a fee-for-service system (25, 29, 30). Therefore, the lack of transfers to healthcare institutions capable of providing intensive rehabilitation during the subacute stage cannot be explained by financial constraints but instead reflects dysfunction in the referral system between healthcare institutions (16). These findings underscore the urgent need for a specialized rehabilitation hospital system that designates eligible conditions and specifies the appropriate timing for inpatient rehabilitation admission.

Examination of the factors influencing LOS revealed that a lower health insurance premium was significantly associated with a longer LOS when the inpatient healthcare institution variable was not included in the model (Supplementary Table S3). However, this association was no longer significant after including the inpatient healthcare institution variable (Table 3). Similarly, older age was significantly associated with a longer LOS when the inpatient variable was excluded; however, this association disappeared when the inpatient variable was added. The Mann–Whitney U and Kruskal–Wallis tests indicated that both health insurance premium and age were associated with inpatient healthcare institutions; however, neither variable exhibited multicollinearity. In addition, the model that included the inpatient healthcare institution variable had lower AIC and BIC values than the model without it. These findings suggest that while individual variables such as health insurance premium and age initially appeared to significantly affect LOS, their influence became non-significant when a more dominant variable, the type of inpatient healthcare institution, was included in the model. Therefore, the type of healthcare institution to which a patient is admitted following acute treatment may have a more significant impact on LOS than health insurance premium or age.

A strength of this study was the use of national-level NHIS-NHID data, which allows the findings to be considered representative of the Korean population. Second, we examined the healthcare delivery system before the introduction of the specialized rehabilitation hospital system, making it the first to explore rehabilitation trends prior to its implementation for patients with TBI in Korea. In addition, the study’s research design may serve as a valuable reference for other countries seeking to implement specialized rehabilitation hospital systems and evaluate their effectiveness.

This study had certain limitations. First, the NHIS-NHID comprises administrative claims data and does not include clinical information such as disease severity or functional recovery (21, 22). We acknowledge that such clinical information may influence the choice of healthcare institution after acute treatment; however, it was not available for this analysis. Second, data on patients’ family composition were limited. Because this family structure is known to influence LOS, this factor may have affected the LOS observed in our study (31).

Third, the South Korean government provides medical expense support to Medical Aid beneficiaries. Type 1 beneficiaries are exempt from inpatient out-of-pocket payments, whereas Type 2 beneficiaries pay only 10% of these costs. Due to this lower financial burden, Medical Aid beneficiaries may have relatively longer LOS than health insurance subscribers (32); therefore, this group was excluded from the analysis. Lastly, to evaluate outcomes for individuals who experienced TBI in 2021, after the introduction of the specialized rehabilitation hospital system, data with a 2-year follow-up would be required, which are not yet available. Therefore, this study should be regarded as a baseline investigation. Once future data become accessible, the findings can be compared with outcomes observed after the introduction of the specialized rehabilitation hospital system. We anticipate that, following implementation, admissions for rehabilitation during the subacute stage—particularly to acute rehabilitation hospitals—will increase.

In conclusion, we characterized the use of the rehabilitation healthcare delivery system among patients with moderate-to-severe TBI prior to the introduction of the specialized rehabilitation hospital system in South Korea. Appropriate transfers to rehabilitation institutions capable of providing intensive rehabilitation after acute TBI treatment were not adequately implemented, as reflected by the 13% transfer rate in the study cohort. A key contributing factor was the absence of formal referral protocols. These findings underscore the need to establish a specialized rehabilitation hospital system.

## Data Availability

The original contributions presented in the study are included in the article/Supplementary material, further inquiries can be directed to the corresponding authors.
