# Supplementary material for: Healthcare utilization for inpatient rehabilitation among people with traumatic brain injury before the introduction of the specialized rehabilitation hospital system: a nationwide retrospective cohort study
Source: Front Neurol. 2025 Nov 21;16:1674199. doi: 10.3389/fneur.2025.1674199 (PMC12678134; doi:10.3389/fneur.2025.1674199)
Supplement: Supplementary file 1 [file Table_1.DOCX]

Supplementary Material

# Supplementary Tables

**Supplementary Table 1.** Length of hospital stay at first healthcare institution (day)

| **Total** | | **Non-inpatient**  **rehabilitation group** | | **Inpatient**  **rehabilitation group** | |
| --- | --- | --- | --- | --- | --- |
| **Mean (SD)** | **Median**  **(Q1–Q3)** | **Mean (SD)** | **Median**  **(Q1–Q3)** | **Mean (SD)** | **Median**  **(Q1–Q3)** |
| 11.9 (18.1) | 8 (3–15) | 10.6 (15.5) | 7 (2–14) | 20.7 (28.9) | 12 (5–25) |

**Supplementary Table 2.** Number of patients by type of healthcare institution at each hospitalization stage

|  | **1st admission** | | **2nd admission** | | **3rd admission** | | **4th admission** | | **5th admission** | |
| --- | --- | --- | --- | --- | --- | --- | --- | --- | --- | --- |
|  | ***N*** | **%** | ***N*** | **%** | ***N*** | **%** | ***N*** | **%** | ***N*** | **%** |
| Total | 27,700 | 100.0 | 3613 | 100.0 | 934 | 100.0 | 364 | 100.0 | 175 | 100.0 |
| Tertiary hospital | 10,920 | 39.4 | 568 | 15.7 | 73 | 7.8 | 24 | 6.6 | 10 | 5.7 |
| General hospital | 16,780 | 60.6 | 975 | 27.0 | 147 | 15.7 | 38 | 10.4 | 10 | 5.7 |
| Primary hospital | 0 | 0.0 | 911 | 25.2 | 243 | 26.0 | 102 | 28.0 | 55 | 31.4 |
| Long-term care hospital | 0 | 0.0 | 993 | 27.5 | 451 | 48.3 | 196 | 53.8 | 96 | 54.9 |
| Clinic | 0 | 0.0 | 166 | 4.6 | 20 | 2.1 | 4 | 1.1 | 4 | 2.3 |

**Supplementary Table 3**. Time required for transfer to a primary hospital and long-term care hospital within 90 days after TBI (*N*=3,613)

|  | ***N*** | **%** | **Time (day)** |
| --- | --- | --- | --- |
| Primary hospital or long-term care hospital | 1,748 | 48.4 | 26.3 |
| Primary hospital | 868 | 24.0 | 21.9 |
| Long-term care hospital | 880 | 24.4 | 30.7 |

**Supplementary Table 4.** Negative binomial regression model for the length of hospital stay in the inpatient rehabilitation group (*N*=3,613)

|  | **Length of stay (day)** | | | |
| --- | --- | --- | --- | --- |
|  | **Adjusted model*** | | | |
|  | **β** | **EXP(β)** | **SE** | **P-value** |
| Age |  |  |  |  |
| <65 | 1.00 |  |  |  |
| 65–74 | 0.26 | 1.29 | 0.06 | <.0001 |
| 75–84 | 0.44 | 1.55 | 0.06 | <.0001 |
| ≥85 | 0.84 | 2.31 | 0.09 | <.0001 |
| Sex |  |  |  |  |
| Male | 1.00 |  |  |  |
| Female | -0.07 | 0.93 | 0.05 | 0.122 |
| Health insurance premium |  |  |  |  |
| First quartile (lowest) | 0.20 | 1.22 | 0.06 | <0.001 |
| Second quartile | 0.15 | 1.17 | 0.06 | 0.009 |
| Third quartile | -0.03 | 0.97 | 0.05 | 0.524 |
| Fourth quartile (highest) | 1.00 |  |  |  |
| Charlson comorbidity index |  |  |  |  |
| Low (0–1) | 1.00 |  |  |  |
| High (≥2) | 1.21 | 3.36 | 0.05 | <0.001 |
| Intensive care unit admission |  |  |  |  |
| Yes | 0.78 | 2.19 | 0.07 | <0.001 |
| No | 1.00 |  |  |  |

*Statistically estimated from negative binomial regression analyses adjusted for all explanatory variables (without inpatient healthcare institutions)
